# Contextual Cueing Effect Under Rapid Presentation

**DOI:** 10.3389/fpsyg.2020.603520

**Published:** 2020-12-16

**Authors:** Xiaowei Xie, Siyi Chen, Xuelian Zang

**Affiliations:** ^1^College of Education, Institutes of Psychological Sciences, Hangzhou Normal University, Hangzhou, China; ^2^Experimental Psychology, Department of Psychology, LMU Munich, Munich, Germany; ^3^Center for Cognition and Brain Disorders, Affiliated Hospital of Hangzhou Normal University, Hangzhou, China; ^4^Zhejiang Key Laboratory for Research in Assessment of Cognitive Impairments, Hangzhou, China

**Keywords:** spatial attention, contextual cueing, visual search, rapid presentation, long term memory

## Abstract

In contextual cueing, previously encountered context tends to facilitate the detection of the target embedded in it than when the target appears in a novel context. In this study, we investigated whether the contextual cueing could develop at early time when the search display was presented briefly. In four experiments, participants searched for a target T in an array of distractor Ls. The results showed that with a rather short presentation time of the search display, participants were able to learn the spatial context and speeded up their response time overall, with the learning effect lasting for a long period. Specifically, the contextual cueing effect was observed either with or without a mask after a duration of 300-ms presentation of the search display. Such a context learning under rapid presentation could not operate only with the local context information repeated, thus suggesting that a global context was required to guide spatial attention when the viewing time of the search display was limited. Overall, these findings indicate that contextual cueing might arise at an “early,” target selection stage and that the global context is necessary for the context learning under rapid presentation to function.

## Introduction

Despite a large amount of information that we experience every day, we have acquired the ability to learn the regularities from the environment. Chun and Jiang (1998) introduced a contextual cueing task, which proved to be a powerful tool to scrutinize the processes involved in environmental statistical learning. In their seminal study, Chun and Jiang (1998) asked the observers to perform a visual search task in which they had to discriminate the direction of a “T”-shaped item target embedded in a set of “L”-shaped distractor items as fast and as accurately as possible. The “context” is defined by the spatial arrangement of distractors. Two types of displays, repeated and novel contexts (sometimes referred to as old and new contexts, respectively) were presented. In the repeated context, there was a stable relationship between the target and distractor locations, which was repeated across the experimental blocks. The novel context on the other hand was used as a control condition, in which distractor locations were determined randomly in every trial and their spatial locations could not predict the target location. It has been widely shown that search speed in the repeated context is faster than novel context (e.g., Chun and Jiang, 1998, 1999; Zheng and Pollmann, 2019; Vadillo et al., 2020b), leading to the suggestions that participants, through repeated encounters of old displays, form some incidental memory about the invariant spatial target-distractor relations in these displays, with this spatial context memory subsequently guiding selective attention more effectively towards the target location. Chun and Phelps (1999) proposed that context memory stores spatial/configural or more general relational information, independent of whether or not this information is acquired implicitly or explicitly. On the other hand, context memory differs largely from other forms of explicit memory. For example, (i) it is fast to acquire: five times of repetition, the search displays are enough to produce the contextual cueing effect (Chun and Jiang, 1998); (ii) it exhibits a large capacity, in that observers are able to form context memory for as many as 60 repeated displays (e.g., Jiang et al., 2005); and (iii) it is robust against interference with time and can last at least 10 days (e.g., Van Asselen and Castelo-Branco, 2009). However, the temporal properties of the context memory are seldom investigated. For instance, how early does the contextual cueing occur?

Studies with event-related potential (ERP) methods indicate that context may be learned within a rather short time (Olson et al., 2001; Johnson et al., 2007; Schankin and Schubo, 2009, 2010). For instance, Johnson et al. (2007) employed the typical contextual cueing paradigm in which participants were asked to detect the target “T” and discriminate its orientation among the distractor “Ls.” They observed a facilitation in reaction times (RTs) for repeated relative to novel contexts, accompanied by an increase in the amplitude of the N2pc waveform beginning at approximately 200 ms post-stimulus. N2pc component is defined as the difference in amplitude between the electrode sites contralateral and ipsilateral to the target, which is a well-validated electrophysiological signature of the focusing of attention (Luck et al., 1997). The difference between repeated and novel contexts in the N2pc component provides direct evidence that contextual cueing leads to greater probability of attention being directed to the visual hemisphere containing the target (see also Schankin and Schubo, 2009). Thus, around 200 ms after the search context, participants could already take use of the learned contextual information to guide attention to the target location. Earlier time differences were also observed with magnetoencephalography (MEG), which showed greater gamma activity to occur 100–300 ms earlier in the repeated than novel conditions (Chaumon et al., 2008).

Although these neurophysiological studies demonstrate relatively rapid emergence of the contextual cueing effect, these findings have seldom been corroborated in behavioral work. In the classic study by Chun and Jiang (1998), the stimulus display was presented until participants responded to the target item, which enabled participants to have an effective connection between the target and (old) contexts. In the subsequent test phase, the stimulus display was presented for 200 ms only (in Experiment 5). The results showed that context memory could be successfully extracted and influence the behavioral response within 200 ms. Note that in this study, there was enough learning time of the stimulus display during the learning stage as the presentation time of the search display was unlimited. Thus, it remains unknown if it is possible to learn the context-target association when the search display is presented with only a limited time. In the “pop-out” search, the characteristics of the single target (such as color and orientation) are different from those of the interference stimuli, and thus participants directed their attention to the target location through bottom-up processing, responding to the target efficiently. The evidence from “pop-out” visual search showed that response could be executed around 600 ms (vs. ca. 1,300 ms in the classical contextual cueing studies by Chun and Jiang, 1998, 2003) after the onset of the search context while also being facilitated by the repeated contextual information (see also Kunar et al., 2008; Geyer et al., 2010). Although they did not separate the learning time from the response time, these findings suggest that it is possible to learn the context within 600 ms of the display time. Moreover, evidence from eye-tracking studies (e.g., Peterson and Kramer, 2001) showed that when participants were performing the standard T/L search task, the probability that the first saccade went to the target on repeated displays was increased relative to novel displays, suggesting that contextual cueing affects behavior as early as the first saccade.

However, it is also possible that contextual cueing could not manifest within a short presentation time. Kunar et al. (2007) presented results that do not support an early onset of the contextual cueing effect. They manipulated the number of search items and measured the search slopes for the repeated and novel configurations but failed to find an improvement in search slopes for repeated over novel display. According to their hypothesis that the visual search slope (reflects response times to the increased number of search items) was assumed as a signature of attentional guidance, a lack of slope difference was interpreted that response-level enhancement but not the early attentional guidance was the reason to driven contextual cueing.

Thus, the goal of the present study was to examine directly if there was an early behavioral gain reflecting the contextual cueing effect. To investigate this question will also help answer the important question whether the context could be learned within a short exposure time. Previous work suggested that contextual cueing cannot be effectively used until search begins (Jiang et al., 2013). Thus, a more straightforward method might be to manipulate the duration of the search display directly. To this end, we investigated whether contextual cueing effects can be acquired under rapid presentation of the search display for the first time to our knowledge.

## Experiment 1

**Figure 1:**
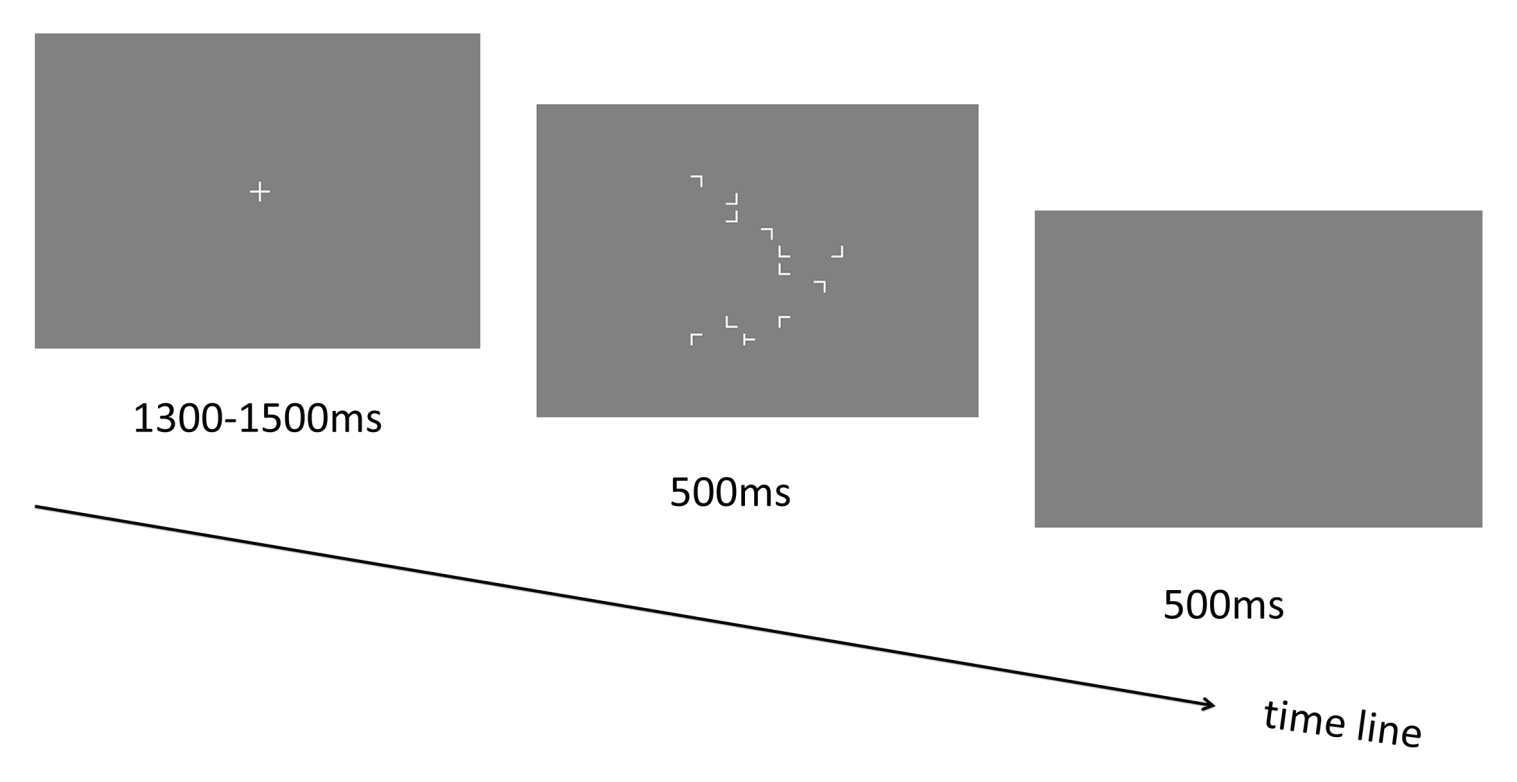
Schematic illustration of the trial sequence in Experiment 1.

To investigate whether the spatial context could affect the target detection with a rapid presentation of the search display, we employed the classical contextual cueing paradigm (Chun and Jiang, 1998) where participants performed visual search for the target object “T” among other distractor objects “Ls” (see Figure 1). Especially, the search contexts were presented for 500 ms only, which is in contrast with previous contextual cueing experiments where the presentation time of the search display was unlimited (Chun and Jiang, 1998). Note that in a previous study with pop-out search paradigm, robust contextual cueing effect was observed, and participants’ mean response times were around 550–650 ms (Geyer et al., 2010). Here, we set the display presentation duration (i.e., 500 ms) being less than the response time threshold that was reported in Geyer et al. (2010) study, to guarantee that no response could be made (i.e., participant could not finish the visual search task) within the display presentation time. The purpose was to exclude the role of response factor in the learning process of contextual cueing.

### Methods

#### Participants

Fifteen naive participants with normal or corrected-to-normal visual acuity (14 females; mean ages: 20.47 ± 1.88 years; all right-handed) were recruited from Hangzhou Normal University. The sample size was estimated by a power analysis using G*Power (Prajapati et al., 2010). In previous contextual cueing tasks, the effect sizes were relatively high (e.g., all *η_p_*^2^s > 0.31 in Makovski and Jiang, 2011; Harris and Remington, 2017; Zhao et al., 2017). Here, we choose a medium effect size (*η_p_*^2^ = 0.25) to estimate the sample size, and the results yielded a sample size of 12 participants per experiment to reach a power of 95% and an *α* level of 0.05. To be more conservative, we recruited 15 participants for each experiment. The study was approved by the ethics committee of the Institutes of Psychological Sciences in Hangzhou Normal University. All participants were given written consent prior to the experiment and were paid ¥50 for their participation.

#### Apparatus and Stimuli

The experiment was conducted in a dimly lit room (ambient light: <1 cd/m^2^). Visual stimuli were presented on a 27-in. LCD monitor (1,920 × 1,080 pixels; 120 Hz). Stimulus presentation and response collection were programmed by using MATLAB and the Psychophysics Toolbox (Brainard, 1997; Pelli, 1997) on an ASUS computer. The distance between the eyes and the computer screen was about 54 cm, with participants’ head position fixed by a chin rest. The background color was gray (luminance: 11.58 cd/m^2^), and the stimulus presentation area was divided into 10 × 10 invisible matrix grid (subtending 12.53° × 12.53° of visual angle). Search items (subtending 0.85° × 0.85° of visual angle) appeared in 12 of the 100 square units, including 11 “L”-shaped distractors (rotated 0, 90, 180, or 270°) and 1 “T”-shaped target rotated 90° to the left or right. The stimuli were presented in white (luminance: 43.34 cd/m^2^). The number of items and the possibility of the target location are equal in each of the four quadrants of the whole stimulus presentation area. The target never appeared in the central 2 × 2 units to prevent participants from looking at the target immediately after the display onset, as the participants were instructed to fix the central cross before the display presentation. In addition, 24 locations on the four corners (each containing six locations) were not used for target’s locations to avoid extreme difficulty in the search task.

#### Design and Procedure

The experiment contained 50 blocks, with 24 trials (12 repeated and 12 novel contexts) in each block, and participants can take a short break every two blocks. The trials with repeated and novel configurations were intermixed randomly in each block. Twelve repeated configurations were randomly generated at the beginning of the experiment and repeated across blocks, whereas 12 novel configurations were newly re-generated in each block. That is, for each repeated context, the locations of the target and distractors, as well as distractors’ orientations (but not the target’s orientations), kept constant throughout the experiment. For the novel context, except for the target’s location (which was constrained to appear at a fixed location in each configuration), both distractors’ locations and orientations varied randomly at each presentation. The orientation of the target (left vs. right) was chosen randomly for each repeated and novel context to avoid potential learning of the targets’ features in visual search.

Each trial started with a central “+” fixation display lasting for 1,300–1,500 ms. Then the visual search display including target and distractors was presented for 500 ms. Participants were asked to respond to the target stimulus “T” as fast and accurately as possible by pressing the response keys (left and right arrow keys for the “T” that is tilted to left and right, respectively). Following the search display, a blank screen was presented for another 500 ms (see Figure 1). Participants could respond during the presentation time (1 s in total) of both the search display and the blank screen to make the response execution as fast as possible (based on a pilot experiment in which we found that most of the responses could be made within 800 ms). Before the start of the experiment, participants were required to perform a practice session including two blocks of 24 trials each (12 repeated and 12 novel). The stimulus displays were presented for 2,500 and 500 ms (same as the training session) in the two blocks to help participants get familiar with the task gradually (starting from an easy condition and then to a difficult condition). Note that all displays used in the practice session were never reused during the experimental phase. Most importantly, participants were not informed in any way that the spatial layout of some trials would be repeated, nor were they told to memorize the display layout.

### Results

In order to improve the power of statistical analysis, every five blocks were collapsed into one epoch for statistical analysis, resulting in 10 epochs in total. Trials with empty or wrong responses were treated as error trials and were not included in the RT analysis. A repeated-measures analysis of variance (ANOVA) with the within-subject factors context (repeated and novel) and epoch (1–10) was conducted on the error rates and RTs. Greenhouse analysis was used when the sphericity of Mauchly’s test was violated. The same analysis was applied to all the subsequent experiments. In addition, Bayes factors (*BF*s) were computed for those results that favored the null hypothesis using JASP software (Marsman and Wagenmakers, 2016). In the calculation process, the default Cauchy settings (i.e., *r*-scale fixed effects = 0.5, *r*-scale random effects = 1, *r*-scale covariates = 0.354) and Cauchy prior (scale = 0.707) were used in the ANOVA and *t*-test, respectively, to calculate *BF*. Specifically, *BF*_10_ was reported to indicate the extent to which the data support the alternative hypothesis (i.e., *H*_1_) as compared with the null hypothesis (*H*_0_). A *BF* value larger than three is considered to provide substantial evidence for alternative hypothesis, while a *BF* less than 1/3 indicates substantial evidence for the null hypothesis (Wetzels et al., 2011).

#### Error Rate

**Figure 2:**
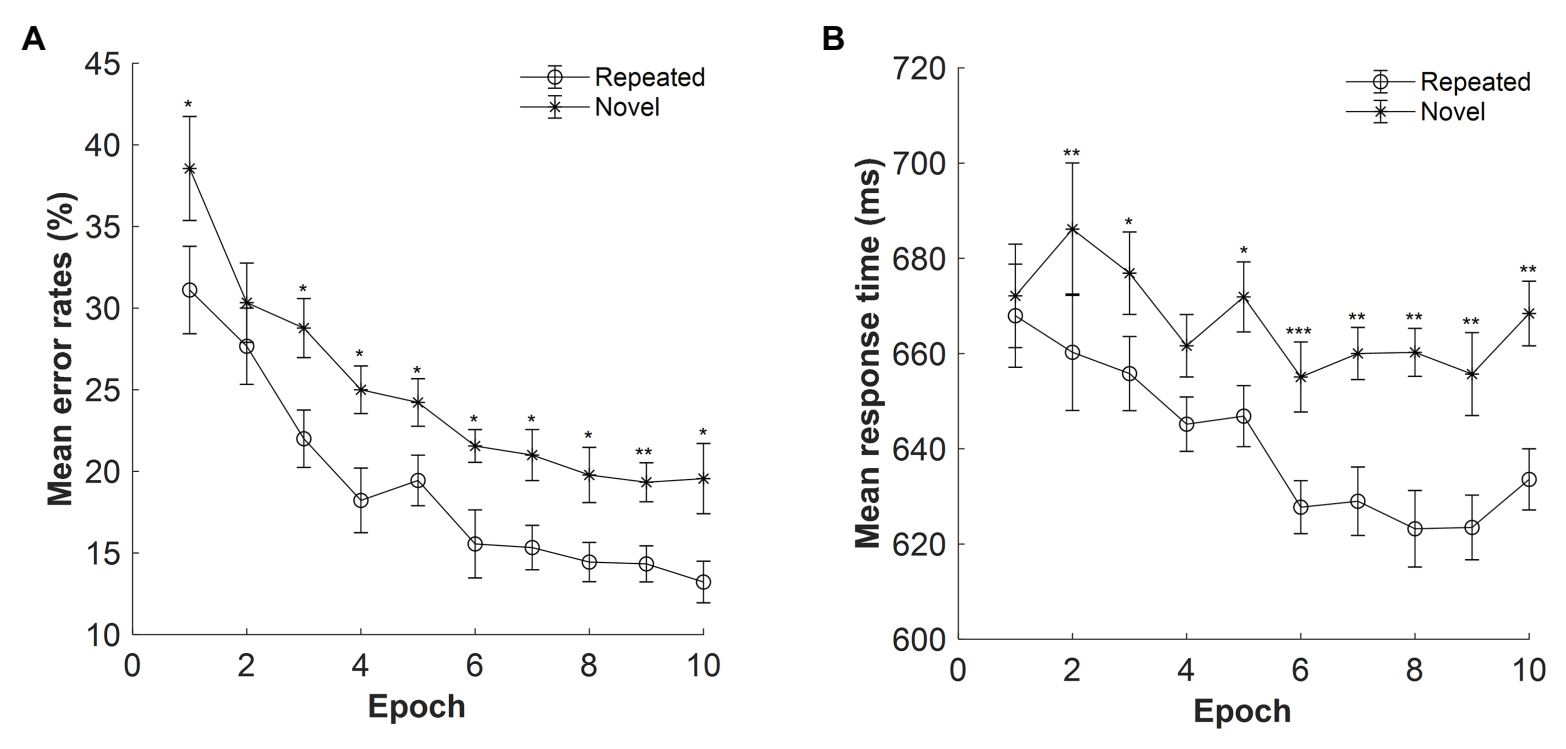
**(A)** Mean error rates as a function of epoch (1–10) and context (repeated, novel) in Experiment 1. **(B)** Mean response time (RT) as a function of epoch and context in Experiment 1. The error bars represent the within-subject standard error of the mean. The star line indicates the novel context, and the circle line the repeated context. Asterisks represent significance levels of *p* < 0.001 (^***^), *p* < 0.01 (^**^), and *p* < 0.05 (^*^).

The mean error rates were high: M = 22%, SE = 0.70%. Figure 2A displays the mean error rates as a function of epoch (1–10) and context (repeated, novel). The results showed a significant main effect of context, *F*(1, 14) = 12.639, *p* = 0.003, *η_p_*^2^ = 0.474, with lower error rates for the repeated context than for the novel context (mean difference: 5.68%), confirming a contextual facilitation in terms of accuracy performance. Thus, participants’ response on the repeated context was more accurate than that on the novel context. The main effect of epoch was also significant, *F*(3.341, 46.771) = 16.076, *p* < 0.001, *η_p_*^2^ = 0.535, with the error rates decreased from 34.83% in Epoch 1 to 16.39% in Epoch 10 (mean difference: 18.44%), suggesting improved performances along with the progress of experiment. The context × epoch interaction was not significant, *F*(9, 126) = 0.584, *p* = 0.808, *η_p_*^2^ = 0.04, *BF*_10_ = 0.013.

#### Reaction Time

Figure 2B depicts the mean RTs for repeated and novel contexts as a function of epoch with the presentation time of 500 ms. The overall mean RTs were 654.06 ms, SE = 20.68 ms. Repeated-measures ANOVA result showed a significant main effect of context, *F*(1, 14) = 17.626, *p* = 0.001, *η_p_*^2^ = 0.557, with a mean cueing effect of 26 ms (mean RT: 641 and 667 ms for the repeated and novel displays, respectively). The main effect of epoch was marginally significant, *F*(2.601, 36.410) = 2.865, *p* = 0.057, *η_p_*^2^ = 0.170, *BF*_10_ = 133.666, with 19 ms faster in Epoch 10 compared with Epoch 1 (651 and 670 ms of Epochs 10 and 1, respectively). The interaction between context and epoch was also significant, *F*(9, 126) = 2.495, *p* = 0.012, *η_p_*^2^ = 0.151. Further *post hoc* analysis showed that the difference between repeated and novel contexts was significant in all epochs (all *t*s > 2.292; all *p*s < 0.038, Cohen’s *d*s > 0.592) except for Epoch 1 [*t*(14) = 0.775, *p* = 0.452, Cohen’s *d* = 0.200, *BF*_10_ = 0.341] and Epoch 4 [*t*(14) = 1.764, *p* = 0.100, Cohen’s *d* = 0.455, *BF*_10_ = 0.914], suggesting that contextual cueing effect was rather stable at the late stage of the experiment.

### Discussion

Experiment 1 showed that under a presentation time of 500 ms, the error rates were lower and RTs were faster in the repeated compared with novel contexts, suggesting that the learning of the spatial context can facilitate the target detection even when the search display was presented for a relatively short time. Moreover, there was a main effect of epoch for both error rates and speed, indicative of procedural learning as the experiment progressed (e.g., Shiffrin and Schneider, 1977). Note that the response speed (mean RT = 654 ms) was much faster when the response time was limited within 1 s, compared with the mean RT (more than 1 s) in previous similar studies with unlimited presentation time of the search display and unlimited response time (i.e., the search display remained on the screen until the response; see, e.g., Chun and Jiang, 1998, 1999), which indicates that responses could be speeded when setting the response boundaries. However, it seems that limiting the response time also made the task more difficult. The initial error rates were rather high, but the accuracy could be greatly improved after a period of training (error rates decreased from 35 to 16%). Given that 500-ms presentation time is sufficient for learning the contextual information with mean RTs around 600–700 ms, it is thus possible that participants could already encode and extract the contextual information before 500 ms. Next, we reduced the presentation time to 300 ms to examine if the contextual cueing effect could also occur.

## Experiment 2

In Experiment 2, we further investigated whether the repeated spatial context could be learned when limiting the presentation time to 300 ms (which is approximately a duration of one fixation; Peterson et al., 2001; Zhao et al., 2012). To this end, we changed the presentation time of the search display to 300 ms while keeping other properties the same as in Experiment 1. In addition, we also provided three additional test sessions with the presentation time prolonged to 2,500 ms after the 10-epoch learning session, in order to test whether the contextual memory learned based on rapidly presented displays could be transferred to normally presented displays (with longer presentation duration) and whether the search difficulty could be reduced when the presentation time was longer. The test sessions were conducted at three time points: right after training, 1 day after training, and 1 week after training.

### Method

**Figure 3:**
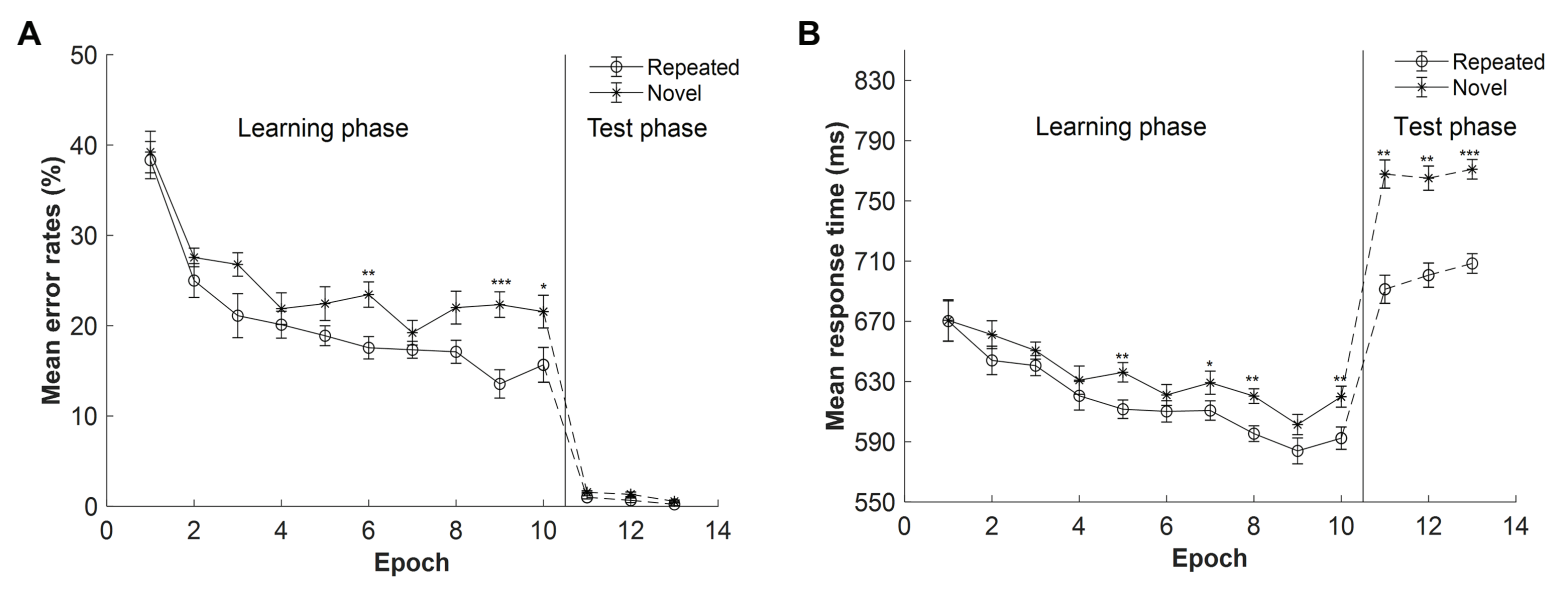
**(A)** Mean error rates as a function of epoch and context in Experiment 2. **(B)** Mean response time (RT) as a function of epoch and context in Experiment 2. The error bars represent the within-subject standard error of the mean. The star line indicates the novel context, and the circle line the repeated context. The solid lines denote the learning phase, and the dashed lines the test phase. Asterisks represent significance levels of *p* < 0.001 (^***^), *p* < 0.01 (^**^), and *p* < 0.05 (^*^).

A new group of 15 participants (13 females; mean ages: 20.2 ± 1.9 years; all right-handed) took part in the experiment. The stimuli, design, and procedure in Experiment 2 were essentially the same as those in Experiment 1 except that the visual search display in the learning session (including 10 epochs of five blocks each) was presented for 300 ms and then the blank screen was presented for 700 ms. In addition, three test sessions with five blocks of 24 trials each were conducted right after training (see Figure 3, Epoch 11: Blocks 51–55), 1 day after training (Epoch 12: Blocks 56–60) and 1 week after training (Epoch 13: Blocks 61–65). Thus, in total, each participant received 1,200 trials in the learning phase and 360 trials in the test phase. The randomly generated new configurations in the last 15 blocks during the learning session (i.e., Blocks 35–50) were reused in the 15 test blocks. In other words, the configurations of Blocks 35–50 in the learning session were identical to those in the three test sessions to control the possible confound resulting from learning when only the repeated context (but not the novel context) was repeated in the test phase. The duration of the search display in the test sessions was extended to 2,500 ms, and the blank screen was 500 ms.

### Result

#### Error Rate

##### Learning Phase

The mean error rates for repeated and novel displays as a function of epoch in the learning session are shown in Figure 3A (Epochs 1–10). When the presentation time of the search display was 300 ms, the mean error rates (M = 23%, SE = 3.25%) were comparable with those in Experiment 1 with 500-ms presentation time [M = 22%, SE = 2.04%, *t*(28) = 0.152, *p* = 0.880, Cohen’s *d* = 0.055, *BF*_10_ = 0.347]. A repeated-measures ANOVA with the factors context (repeated, novel) and epoch (1–10) showed that main effects of both context and epoch were significant: context, *F*(1, 14) = 10.198, *p* = 0.007, *η_p_*^2^ = 0.421, with lower error rates for the repeated than novel context (20.47 and 24.64%, respectively, mean difference = 4.17%); and epoch, *F*(3.150, 44.104) = 23.461, *p* < 0.001, *η_p_*^2^ = 0.626, with a decrease of 20.17% from Epoch 1 (38.78%) to Epoch 10 (18.61%). There was a marginally significant interaction between context and epoch, *F*(9, 126) = 1.742, *p* = 0.086, *η_p_*^2^ = 0.111, *BF*_10_ = 0.084. The *post hoc* analysis showed that the difference between repeated and novel contexts in the error rates reached significance in Epoch 6, Epoch 9, and Epoch 10 (all *t*s > 2.685, all *p*s < 0.018, Cohen’s *d*s > 0.693).

##### Test Phase

In the three test sessions, participants were given enough search time (i.e., 2.5 s), which greatly decreased the error rates as compared with the learning session (see Figure 3A, Epochs 11–13): M = 0.89%, SE = 0.13%, *t*(14) = 6.670, *p* < 0.001, Cohen’s *d* = 1.722. A repeated-measures ANOVA with the factors context (repeated, novel) and epoch (11–13) showed significant main effects of both context and epoch: context, *F*(1, 14) = 5.237, *p* = 0.038, *η_p_*^2^ = 0.272, with the mean error rates lower for the repeated than novel context (mean difference = 0.52%, SE = 0.23%); and epoch, *F*(2, 28) = 4.207, *p* = 0.025, *η_p_*^2^ = 0.231, with mean error rates decreased from Epoch 11 to Epoch 13 (mean difference = 0.89%, SE = 0.31%). The context × epoch interaction was not significant, *F*(2, 28) = 0.183, *p* = 0.834, *η_p_*^2^ = 0.013, *BF*_10_ = 0.184.

#### Reaction Time

##### Learning Phase

The mean RTs for repeated and novel contexts as a function of epoch in the learning session are depicted in Figure 3B (Epochs 1–10). The mean RTs were comparable for the 500- and 300-ms presentation time conditions (654 vs. 626 ms), *t*(28) = 0.956, *p* = 0.347, Cohen’s *d* = 0.349, *BF*_10_ = 0.486. A repeated-measures ANOVA of RTs with 300-ms presentation with factors context (repeated and novel) and epoch (1–10) showed significant main effects of context, epoch, and context by epoch interaction: context, *F*(1, 14) = 7.327, *p* = 0.017, *η_p_*^2^ = 0.344, with a mean cueing effect of 16 ms (618 and 634 ms for repeated and novel context, respectively); epoch, *F*(3.262, 45.670) = 9.869, *p* < 0.001, *η_p_*^2^ = 0.413, with RTs decreased of 64 ms from Epoch 1 (670 ms) to Epoch 10 (606 ms); context × epoch interaction, *F*(9, 126) = 2.097, *p* = 0.034, *η_p_*^2^ = 0.130, indicating that the contextual cueing effect increased along with the progress of experiment. In addition, the cueing effect (i.e., RT_novel_ − RT_repeated_) was comparable with that in Experiment 1 with 500-ms presentation time (26 ms), *t*(28) = 1.094, *p* = 0.283, Cohen’s *d* = 0.400, *BF*_10_ = 0.541.

##### Test Phase

The mean RTs in the test session are depicted in Figure 3B (Epochs 11–13). A repeated-measures ANOVA with factors context (repeated, novel) and epoch (11–13) showed a significant main effect of context, *F*(1, 14) = 25.761, *p* < 0.001, *η_p_*^2^ = 0.648. The mean cueing effects were of 76.5, 64.4, and 62.5 ms for the three test sessions. The effect of epoch was not significant, *F*(2, 28) = 0.295, *p* = 0.747, *η_p_*^2^ = 0.021, *BF*_10_ = 0.119. And the interaction between context and epoch was not significant, *F*(2, 28) = 0.473, *p* = 0.628, *η_p_*^2^ = 0.03, *BF*_10_ = 0.181. These results suggest that contextual cueing effect occurred in the test sessions and that the amplitudes of contextual cueing effect were comparable for the three sessions.

To further examine whether the contextual cueing effect observed in the test phase was due to new learning effect in the test phase or due to the transfer effect from the previous learning phase, a paired sample *t*-test was applied to compare the difference in RTs between repeated and novel contexts for the first block of each test session, given that the configurations in the first block were all presented once and thus not repeated yet. Moreover, the changes of presentation time (from 300 ms in the training session to 2,500 ms in the test session) would only influence the RT equally for the novel and repeated conditions in the first block of the test session (with comparable properties) but not influence their RT difference (i.e., contextual cueing effect), thus excluding the possible influence of the presentation time on the transfer of context cueing. The results revealed significant contextual cueing effect in the first block of all test sessions, Session 1: *t*(14) = 4.218, *p* = 0.001, Cohen’s *d* = 1.089, mean difference = 116.27 ± 27.57 ms; Session 2: *t*(14) = 2.790, *p* = 0.014, Cohen’s *d* = 0.720, mean difference = 48.12 ± 17.25 ms; Session 3: *t*(14) = 2.712, *p* = 0.017, Cohen’s *d* = 0.700, mean difference = 77.62 ± 28.62 ms, thus indicating that there was a transfer of the context memory from the learning phase to the test phase. Moreover, there was a much larger contextual cueing effect in Block 51 (the first block in the first test session) compared with Block 50 (the last block in the learning session), *t*(14) = 2.727, *p* = 0.016, Cohen’s *d* = 0.704, mean difference = 86 ms, possibly due to longer presentation time in the test phase.

### Discussion

Experiment 2 showed that there were significant differences in error rates and RTs between repeated and novel contexts in the learning session, replicating the results in Experiment 1. Thus, contextual information can be learned under the rapid presentation of 300 ms. Most important, the contextual cueing effect was comparable between Experiments 1 and 2, suggesting that shortening the presentation time of the search display from 500 to 300 ms would not significantly impede contextual learning. Moreover, the contextual memory maintained under rapid presentation time could last as long as 1 week, replicating previous studies to show that contextual cueing effect is a long-term memory effect (Chun and Jiang, 2003; Jiang et al., 2005; Van Asselen and Castelo-Branco, 2009).

Note that when the response time was limited to 1 s, participants tended to make a speeded response around 550–650 ms (in both Experiments 1 and 2), which duration was comparable with the RT in the pop-out search where the search for the salient target is rather efficient (Geyer et al., 2010). In contrast, when the response limitation was changed to 2.5 s (in Experiment 2), the RT was correspondingly extended. In contrast to the RT, the accuracy was greatly dropped when the response limitation was 1 compared with 2.5 s. Thus, it appears that a strategy applying speed-accuracy trade-off was used among participants. It has been shown that searching for a “T” among distractors “L” involves a serial processing (Treisman and Gelade, 1980; Duncan and Humphreys, 1989), which is a demanding search task strongly dependent on the focused spatial attention of the display (Woodman and Luck, 2003). Thus, it is possible that with limited response time, it is more difficult for the participants to correctly localize and identify the target. Despite the increased task difficulty due to the limitation of the response time, we nevertheless found that context information could be learned and extracted to guide the attention more effectively.

Previous eye-tracking studies showed that the first saccade on average landed already closer to the target for the repeated configurations than new configurations (e.g., Peterson and Kramer, 2001) with an average fixation duration of up to 300 ms (Peterson et al., 2001; Zhao et al., 2012). Given attention can be guided to the general vicinity of the target for the initial fixations (Peterson and Kramer, 2001), it is possible that the contextual memory only relies on the local context of the target within a rather short time of viewing. There is evidence that local invariances are important for successful contextual learning (Olson and Chun, 2002; Song and Jiang, 2005; Brady and Chun, 2007). For instance, Brady and Chun (2007) showed that when the repeated distractors (e.g., 2 “Ls”) were locally positioned near the target, participants were able to acquire the context in the learning phase, suggesting that near-target invariant inter-element relations are important for contextual learning. However, other studies showed that the acquired cueing effects transferred from the learning to the test session only for search displays that maintained the global information, but not for displays that only maintained the local set of objects near the target (Brockmole et al., 2006), supporting the important role of global context in contextual learning and transfer (Kunar et al., 2006; Geyer et al., 2010; Rosenbaum and Jiang, 2013). More recent evidence showed that effective retrieval for search guidance required the availability of peripheral information (Zang et al., 2015). In the next experiment, we set out to solve the question of whether information learned within 300-ms viewing time is global or local context.

## Experiment 3

Experiment 2 showed that contextual cueing effect could be effectively observed with the search display presented for 300 ms. However, it is unclear how the contextual information could be learned and extracted when it is only available for a rather short time. Experiment 3 investigated whether the learning and retrieval of invariant display properties require global structure of the context or whether the availability of the local structure of the context is sufficient for the contextual cueing to manifest. To this end, in Experiment 3, we changed the repeated configurations so that only the local layouts in which two distractor items within the target quadrant were repeated across blocks whereas the remaining distractor items in other quadrants were located randomly across trials (see also Brady and Chun, 2007). The novel configurations were generated randomly across trials, which were similar to those of Experiments 1 and 2. If contextual cueing relies on a global context, we would observe a null finding. However, if the local layout of the configuration is sufficient to guide attention to the target, we would observe a contextual cueing effect.

### Method

**Figure 4:**
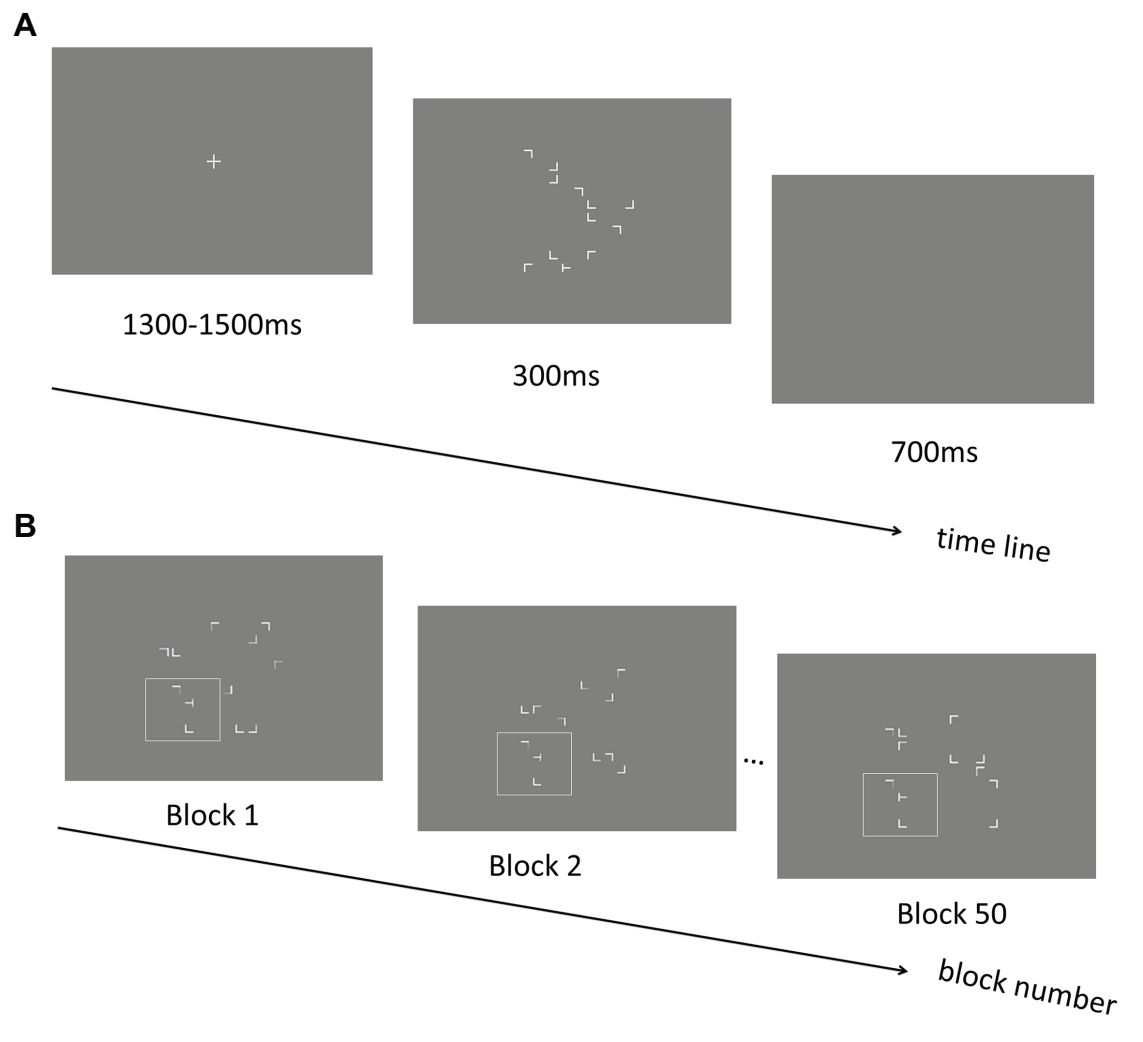
**(A)** Schematic illustration of the trial sequence in Experiment 3. **(B)** An example of repeated display in the Experiment 3. The spatial relationship between the positions of the distractors and the target within the quadrant containing the target is preserved across blocks (illustrated as letters in the white box, which was not shown in the real experiment). The positions of the remaining distractor stimuli are generated randomly across blocks.

A new group of 15 participants (13 females; mean ages: 21.40 ± 0.46 years; all right-handed) took part in the experiment. The stimuli, design, and procedure in Experiment 3 (see Figure 4A) were essentially the same as those in the training phase of Experiment 2 except that in the repeated configurations, only the local layouts (i.e., two distractors and one target) were repeated across blocks whereas the spatial locations of distractors in the other three quadrants were randomly manipulated across trials (see Figure 4B). In addition, each of the four quadrants had equal possibility of the local repeated configurations. In the local layout, the target and two near distractors were presented within a view window sized 6.27° × 6.27°, whereas the whole stimulus presentation area (with 12.53° × 12.53° of visual angle) was kept the same as previous experiments.

### Result

#### Error Rate

**Figure 5:**
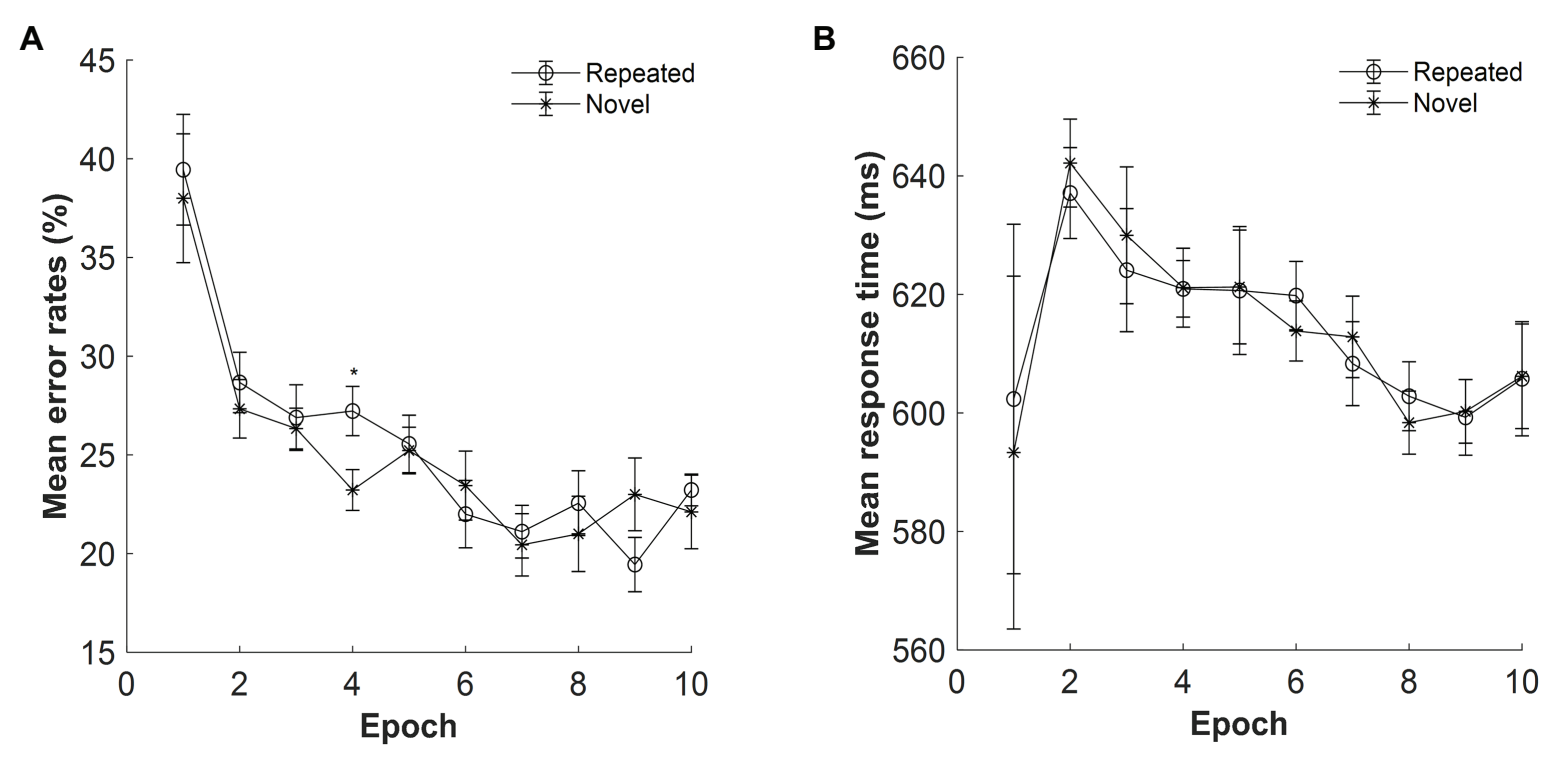
**(A)** Mean error rates as a function of epoch and context in Experiment 3. **(B)** Mean response time (RT) as a function of epoch and context in Experiment 3. The error bars represent the within-subject standard error of the mean. The star line indicates the novel context, and the circle line the repeated context. Asterisks represent significance levels of *p* < 0.05 (^*^).

The mean error rates were high: M = 25%, SE = 2.63%. Figure 5A displays the mean error rates as a function of epoch (1–10) and context (repeated and novel). The error rates were analyzed by repeated-measures ANOVA with the within-subject factors context (repeated and novel) and epoch (1–10). The results showed that the main effect of context was not significant, *F*(1, 14) = 0.327, *p* = 0.577, *η_p_*^2^ = 0.023, *BF*_10_ = 0.153, which suggested that the mean error rates were comparable between the repeated and novel contexts. The main effect of epoch was significant, *F*(3.115, 43.616) = 12.435, *p* < 0.001, *η_p_*^2^ = 0.470, with decreased error rates from Epoch 1 to Epoch 10 (mean difference = 16.06%, SE = 3.62%). The display × epoch interaction was not significant, *F*(9, 126) = 1.377, *p* = 0.205, *η_p_*^2^ = 0.090, *BF*_10_ = 0.032.

#### Reaction Time

The mean RTs for repeated and novel contexts as a function of epoch are shown in Figure 5B. Repeated-measures ANOVA with within-subject factors context (repeated, novel) and epoch (1–10) on the RTs showed that the main effects of both context and epoch were not significant, context: *F*(1, 14) = 0.004, *p* = 0.952, *η_p_*^2^ < 0.001, *BF*_10_ = 0.125; epoch: *F*(1.717, 24.033) = 1.245, *p* = 0.301, *η_p_*^2^ = 0.082, *BF*_10_ = 2.259, with *BF*s indicating that the alternative hypothesis is 2.26 times more likely than that of the null hypothesis. The interaction between context and epoch was not significant, *F*(9, 126) = 0.741, *p* = 0.671, *η_p_*^2^ = 0.050, *BF*_10_ = 0.011. Importantly, when further analyzing RTs from the second epoch, significant main effect of epoch was observed, *F*(3.735, 52.289) = 3.780, *p* = 0.010, *η_p_*^2^ = 0.213, with RTs decreased at 34 ms from Epoch 2 (640 ms) to Epoch 10 (606 ms), indicating the procedural learning effect. This might be due to a different search strategy from Epoch 1 to Epoch 2 as indicated by higher error rates in Epoch 1 (39%) than Epoch 2 [28%; *t*(14) = 3.129, *p* = 0.007, Cohen’s *d* = 0.808]. However, due to the high error rates, participants’ RT response in the first epoch may not provide enough statistic power.

### Discussion

In Experiment 3, the contextual cueing effect was not observed by repeating only the local layout of the repeated configuration (with the remaining distractors randomly distributed across trials) with a presentation time of 300 ms. Note that the size of the stimuli display used in the current study was comparable with that in Zang et al. (2015) study where a view window sized 12° made the peripheral information available and thereby enhanced contextual retrieval. They argued that additional information from the periphery (outside the 8° area) likely contributes to optimizing (online) saccadic path planning, which is important for retrieving the learned spatial inter-element relations from contextual memory. In the present experiment, no contextual cueing effect occurred by repeating the local configuration consisting of just two to three items within a view window sized 6.27° while changing the peripheral information in the rest presentation area. Thus, even with visible peripheral information beyond the local layout, if the peripheral information was not invariant, there was still no context-based search guidance. These results indicate that global contextual information is required for the contextual cueing effect to manifest with a 300-ms viewing time of the search display.

## Experiment 4

Previous experiments showed that context can be learned under a rapid presentation of 300 ms. However, it is also possible that the learning of context also occurs *via* an internal representation after the display disappears due to effects of visual persistence (Coltheart, 1980). In Experiment 4, we employed backward masking of the search displays to limit the processing time to 300 ms. Moreover, we introduced a recognition test at the end of the experiment to examine whether participants had awareness of the repeated configurations.

### Methods

**Figure 6:**
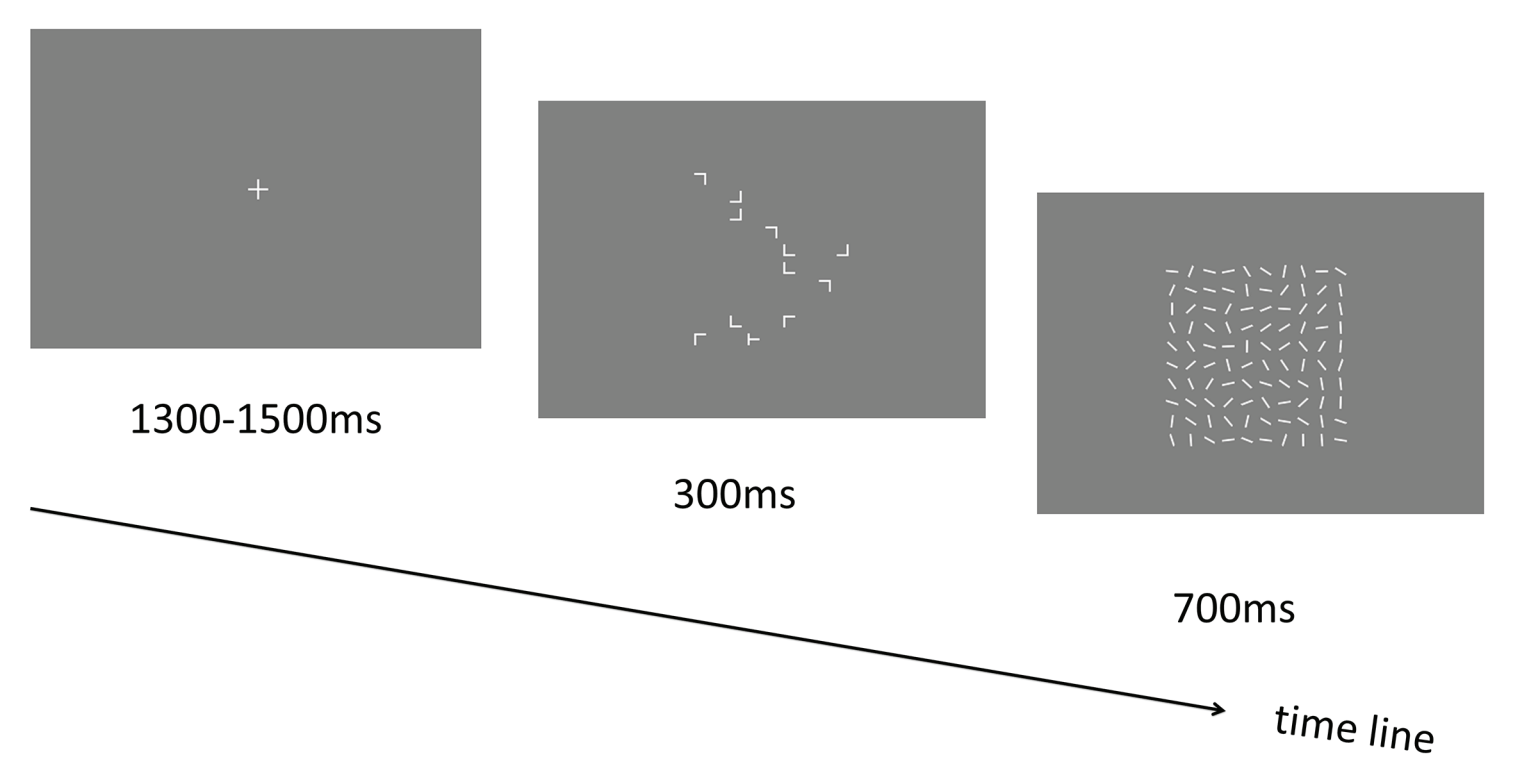
Schematic illustration of the trial sequence in Experiment 4.

In Experiment 4, a new group of 15 participants (10 females; mean ages: 21.07 ± 0.37 years; all right-handed) were tested. Experiment 4 was essentially the same as Experiment 2, except that after the search display disappeared, the blank screen in Experiment 2 was replaced by a mask display presenting for 700 ms (see Figure 6). The masking stimuli were composed of 100 white lines with random orientations presented at each grid of the stimulus presentation area (with 10 × 10 invisible matrix grid; see Experiment 1). Participants could respond to the target “T” after the onset of the search display until the offset of the mask display. The response time was also limited to 1 s (from the onset of the search display until the end of the masking stimuli). After the experiment, a recognition task (with 24 trials) was carried out. Each trial started with a central “+” fixation display lasting for 1,300 to 1,500 ms. Then the visual search display including target and distractors was presented for 2,500 ms. Participants were instructed to press the left arrow key if they feel that they had seen this configuration (i.e., repeated display) in the earlier visual search blocks or the right arrow key if they recognize this configuration as novel display. The repeated displays that had been presented in the earlier search blocks (i.e., 12 displays) and 12 newly generated configurations (that had never appeared before) were randomly intermixed across trials.

### Result

#### Error Rate

**Figure 7:**
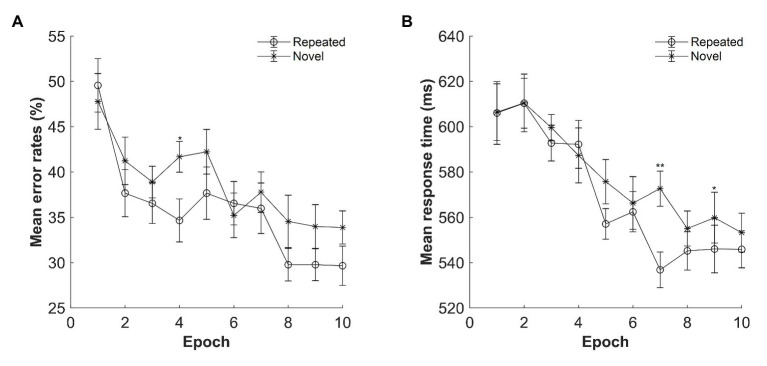
**(A)** Mean error rates as a function of epoch and context in Experiment 4. **(B)** Mean response time (RT) as a function of epoch and context in Experiment 4. The error bars represent the within-subject standard error of the mean. The star line indicates the novel context, and the circle line the repeated context. Asterisks represent significance levels of *p* < 0.01 (^**^) and *p* < 0.05 (^*^).

The mean error rates were high: M = 37.26%, SE = 3.73%. Figure 7A displays the mean error rates as a function of epoch (1–10) and context (repeated and novel). A repeated-measures ANOVA with the factors context (repeated and novel) and epoch (1–10) showed no effect of context, *F*(1, 14) = 2.199, *p* = 0.160, *η_p_*^2^ = 0.136, *BF*_10_ = 1.977, with *BF*s indicating that the alternative hypothesis is 1.97 times more likely than that of the null hypothesis. The main effect of epoch was significant, *F*(2.507, 35.092) = 5.968, *p* < 0.001, *η_p_*^2^ = 0.299, with decreased error rates from Epoch 1 to Epoch 10 (mean difference = 16.89%, SE = 3.21%), suggesting improved performances along with the progress of experiment. The context × epoch interaction was not significant, *F*(9, 126) = 1.815, *p* = 0.072, *η_p_*^2^ = 0.115, *BF*_10_ = 0.031.

#### Reaction Time

The mean RTs for repeated and novel contexts as a function of epoch are shown in Figure 7B. A repeated-measures ANOVA with factors context (repeated and novel) and epoch (1–10) showed only a main effect of epoch, *F*(3.370, 47.179) = 7.211, *p* < 0.001, *η_p_*^2^ = 0.340, with 57 ms faster in Epoch 10 compared with Epoch 1. There was no significant effect of context, *F*(1, 14) = 2.328, *p* = 0.149, *η_p_*^2^ = 0.143, *BF*_10_ = 0.536, or of two-way interaction, *F*(3.387, 47.412) = 1.794, *p* = 0.155, *η_p_*^2^ = 0.114, *BF*_10_ = 0.033. However, when considering the last four epochs only, the same analysis with factors context (repeated, novel) and epoch (7–10) showed a significant main effect of context, *F*(1, 14) = 7.048, *p* = 0.019, *η_p_*^2^ = 0.335, with a mean cueing effect of 16.71 ms, and a significant interaction between context and epoch, *F*(3, 42) = 3.371, *p* = 0.027, *η_p_*^2^ = 0.194, with the largest contextual cueing effect at Epoch 7 compared with other epochs (*p*s < 0.05, Cohen’s *d* > 0.542). The main effect of epoch was not significant, *F*(3, 42) = 0.141, *p* = 0.935, *η_p_*^2^ = 0.010, *BF*_10_ = 0.055. Thus, contextual cueing effect only occurred at the late stage of the learning phase.

Next, we compared the performance between the experiments with (Experiment 4) and without masking stimuli (Experiment 2). To this end, we conducted 2 (experiment: Experiment 2 vs. Experiment 4) × 2 (context: repeated vs. novel) × 10 (epoch: 1–10) mixed ANOVAs, which showed that there was no significant difference between the two experiments: *F*(1, 28) = 2.098, *p* = 0.159, *η_p_*^2^ = 0.070, *BF*_10_ = 0.945, indicating that the mean RTs were comparable between the two experiments (M = 626 ms for Experiment 2 and M = 574 ms for Experiment 4), and any interactions with the factor experiment were all not significant, all *F*s ≤ 1, all *p*s > 0.370, all *η_p_*^2^s < 0.040, all *BF*_10_s < 0.190. An independent-samples *t*-test was further carried out for the contextual cuing effect averaged Epochs 1–10 in Experiment 2 and Experiment 4. The results showed that there was no significant difference in the averaged contextual cueing effect between Experiment 2 (M = 16.18 ms, SE = 5.98 ms) and Experiment 4 (M = 9.17 ms, SE = 6.01 ms), *t*(28) = 0.827, *p* = 0.415, Cohen’s *d* = 0.302, *BF*_10_ = 0.446. Thus, it appears that the amount of contextual cueing effect was comparable regardless if with or without masking stimuli.

#### Recognition Task

We examined participants’ recognition performance by means of the recognition sensitivity d′ [d′ = Z (hit rate) − Z (false-alarm rate) (Green and Swets, 1966)]. A hit means that participants correctly judged a “repeated” configuration as “old,” while a false alarm means that they incorrectly judged a “novel,” random configuration as “old.” The hit and false alarm rates were 53 and 48%, respectively. The mean d′ was 0.13 (SE = 0.19) and not significantly different from zero, *t*(14) = 0.679, *p* = 0.508, Cohen’s *d* = 0.175, *BF*_10_ = 0.321, indicating that participants did not have explicit memory for repeated context.

### Discussion

In Experiment 4, under a rapid presentation time of 300 ms, we employed a procedure of backward masking to block the visual processing after the search displays. The results showed a contextual cueing effect but only occurred at the late stage of the learning. The mean response time and averaged contextual cueing effect under backward masking were comparable with the condition where the internal visual processing was not blocked. Furthermore, post-experimental recognition tests revealed participants’ ability to distinguish repeated (old) from novel (new) conditions only to be at chance level, suggesting that the acquired contextual memory could be implicit. It is important to mention that we have only 24 trials in the recognition session, which may lack enough statistical power to make a firm conclusion, as some studies have discussed the power problems in recognition tests of contextual cueing (see, e.g., Smyth and Shanks, 2008; Vadillo et al., 2016).

## General Discussion

The current study investigated whether the contextual cueing effect could be observed when the search context was presented briefly. Specifically, the search stimuli were presented for 500 ms in Experiment 1 and for 300 ms in Experiment 2, and in Experiment 4 with the search display masked after 300 ms, which showed that participants were able to learn the spatial context within a short presentation time, leading to faster search response for repeated than novel contexts. Moreover, the learning effect acquired under 300-ms presentation time could last as long as 1 week (as shown in Experiment 2), similar to the contextual cueing effect obtained under unlimited presentation time (Chun and Jiang, 2003). In addition, we further showed that such a context learning under rapid presentation required the availability of the global context information instead of the local context information (in Experiment 3). Furthermore, post-experimental recognition tests revealed participants’ ability to distinguish repeated from novel conditions only to be at chance level, indicating that contextual cueing is mediated by implicit memory representations. Taken together, the results provided first evidence that context could be learned and acquired to guide attention effectively within a rather short time.

Previous behavioral studies showed that context memory could be successfully extracted within the 200-ms presentation time in the subsequent test phase after the initial learning phase with unlimited presentation time (Chun and Jiang, 1998). The present study showed that the contextual information could be learned effectively within a limited time as short as 300 ms. This suggests that contextual cueing might occur rather early (before 300 ms) in the search process, which is also supported by the results that the overall search RTs were greatly reduced when the display presentation time was limited to 300 ms compared with when the presentation time was extended to 2,500 ms (in Experiment 2). This finding is in contrast with previous behavioral studies, which suggested a slow time course of contextual cueing (Kunar et al., 2007, 2008). For instance, Kunar et al. (2008) found that search slopes were shallower in the repeated than in the novel condition, but only when the overall search took longer with slowed search RTs; otherwise, there was no difference in the search slopes between the repeated and novel conditions when the number of the search items was varied (Kunar et al., 2007). Therefore, they concluded that the cueing benefits might arise “late” in processing, i.e., at the response selection stage. Instead, the present study provided evidence of behavioral gains at an early time, which is consistent with the neurophysiological indices reflecting that spatial attention diverges as early as 100–200 ms between the repeated and novel displays (e.g., Johnson et al., 2007; Chaumon et al., 2008; Schankin and Schubo, 2009). Accordingly, it is possible that contextual cueing influences an “early,” target selection stage (e.g., Chun and Jiang, 1998; Johnson et al., 2007). That is, contextual cuing arises because observers learn the predictive structure of the search environment by associating the positions of distractors in repeated displays with the location of the target, thus promoting the search efficiency of the task (in line with Chun and Jiang, 1998, attentional-guidance account).

The original contextual cueing paradigm showed that observers implicitly learn the repeated configuration of targets in visual search tasks and that this context can serve to cue the target location and facilitate search performance in subsequent encounters (Chun and Jiang, 1998). Thus, the process of search through distractors to find the target is crucial for contextual cueing (Olson and Chun, 2002). Interestingly, there is evidence that repeating the locations just of items in the target’s quadrant produces as much contextual cueing as does repeating the entire display (Song and Jiang, 2005; Brady and Chun, 2007). In Brady and Chun’s study, only the target adjacent locations were attended and incorporated into learning, suggesting that contextual cueing effect relies on the local context of the target. However, we found that with 300-ms presentation time, only repeating the distractor locations inside the target quadrant was not able to produce contextual cueing effect (in Experiment 3), indicating that the learning of the contextual information presented rapidly did not incorporate local configuration information. Yet by combining the results of Experiments 2 and 4, which revealed a significant contextual cueing effect when the whole display was repeated, we could infer that participants learned the global context and performed a global search mode.

It should be noted that the size of the stimuli presentation area (with visual angle of 12.53° × 12.53°) was much smaller in our study compared with that (the entire screen) in Brady and Chun’s study. Correspondingly, the size of the single stimulus in the present study was also much smaller (0.85° × 0.85° in our study relative to 1.8° × 1.8° in Brady and Chun’s study). The difference in the size of the stimuli presentation area (and of the letters) might be the critical factor that leads to the difference in the search mechanism. That is, with a smaller presentation area of the stimuli that presented briefly, it is easier for the participants to encode the global configuration without the necessity to frequently shift their attention from one stimulus to the other in order to locate the target. Alternatively, given that Brady and Chun (2007) did not limit the presentation time of the search display, it might be possible that provided enough viewing time when participants were able to process the local context, the local context could guide the attention to the target location as well. In line with our findings, Zang et al. (2015) contextual cueing study presented stimuli within a circular display matrix with a diameter of 16° of visual angle, which was nearly comparable with our study and found that repeated contexts could not be effectively retrieved based on the learned local context under limited viewing condition (e.g., only two distractors near the target can be seen) to aid search guidance. However, once (some) peripheral global information was provided or the whole display configuration was previewed, the contextual cueing effect immediately manifested, suggesting that global information is necessary for contextual retrieval.

The observation of a behavioral gain around 300 ms in the present study is in line with previous neurophysiological evidence showing that the N2pc component of the ERP was greater for repeated than for novel displays (Johnson et al., 2007; Schankin and Schubo, 2009). That is, attention could be allocated effectively to the target’s location in repeated context around 200–300 ms after the search display onset as concluded in previous studies (e.g., Schankin and Schubo, 2009). However, it is quite obvious that 200 ms could not guarantee participants to identify the target’s location and direct attention to the target; otherwise, participant’s mean search time should be much shorter than that (more than 1 s) reported in previous studies that did not limit participant’s viewing time (e.g., Chun and Jiang, 1998; Olson and Chun, 2002; Brady and Chun, 2007). Based on our results that 300 ms was sufficient to learn and extract the global configuration of the contextual information, we propose that participants might first process the search display globally and then could direct attention to the local context near the target. Therefore, the N2pc component may indicate attentional guidance to the global search display but not to the exact or near target’s location. This, however, would require further investigation.

To further explain the underlying search mechanism, one possibility is that context learning under rapid presentation requires the associative learning between a global context and the target location with the top-down influence of the integrated representation on attentional guidance (Chun, 2000; Chun and Nakayama, 2000). With effective learning, a perceptual unit that integrates the spatial association of the target and distractors of a display might be extracted and formed. Specifically, the formation and reinforcement of this perceptual unit across repetitions might be accompanied by an enhancement of its visual saliency (Geyer et al., 2010), which captures spatial attention in a bottom-up way by using near-peripheral vision (Zang et al., 2015). This process is also constrained by spatial attention and working memory limitations (Gobet et al., 2001). Based on our results, all the visual stimuli could be simultaneously held in attentional window and thus grouped together, effectively encoded into working memory within 300 ms. These temporally learned configurations then translate to long-term memory along with the learning time, subsequently guiding focal attention to the target location when learned pattern re-occurs on later occasions. Alternatively, recent studies suggest that the learning of the distractor configuration could also facilitate the target detection without the guidance to the target location (Vadillo et al., 2020a). Vadillo et al. (2020a) observed a significant contextual cueing effect in visual search even when the target location cannot be predicted by the distractors in repeated configurations (with the locations of distractors kept constant but the locations of the target changed randomly). They suggested that participants learn to ignore the locations usually occupied by distractors, which in turn facilitates the detection of targets. Accordingly, it is possible that with rapid presentation of the contextual information in the current study, the learning of the global configuration makes the distractors suppressed, and thereby, the target becomes more salient, which facilitates the target detection. In line with this account, Zinchenko et al. (2020) also showed that a broad attentional set facilitates flexible updating of global (relative to local) context representations, making the acquired context memory be more adaptive to the changes of the targets. However, to disentangle the two accounts requires further research.

However, our study had several limitations: first, although our sample size has a good power, it is better to use a larger sample size to increase the generalizability of the effect. Moreover, it might be interesting for future work to use more ecological stimuli (see, e.g., Santangelo, 2015; Santangelo et al., 2015, for a review) to replicate current results. In addition, we only investigated the presentation time of 300 and 500 ms, but 300 ms might be not the minimum presentation time to get a contextual cueing effect, which also requires further research.

To summarize, the present study showed that a long-term context memory could be acquired under a rapid presentation of the search display, suggesting that contextual cueing might arise at an “early,” target selection stage. Moreover, the obtained contextual cueing effect with short presentation time did not result from the learning of repeated local configuration of items, thus indicating that a more global context was required. This novel finding sheds light on the temporal attributes of the contextual cueing effect and provides a possible answer as to the underlying learning mechanism when the presentation time is limited.

## Data Availability Statement

The original contributions presented in the study are included in the article/supplementary material, further inquiries can be directed to the corresponding author. The data files and analysis scripts are available at a public repository https://github.com/Xie-0130/contextual-cueing.

## Ethics Statement

The studies involving human participants were reviewed and approved by the ethics committee of the Institutes of Psychological Sciences in Hangzhou Normal University. The patients/participants provided their written informed consent to participate in this study.

## Author Contributions

SC and XZ are responsible for experimental design, results interpretation, manuscript revision, and final approval. XZ programmed the code for the experiments. XZ and XX collected and analyzed the data. XX and SC drafted the manuscript. All authors agree to be accountable for the content of the work.

### Conflict of Interest

The authors declare that the research was conducted in the absence of any commercial or financial relationships that could be construed as a potential conflict of interest.
